# In Vitro Interactions of Moroccan Propolis Phytochemical’s on Human Tumor Cell Lines and Anti-Inflammatory Properties

**DOI:** 10.3390/biom9080315

**Published:** 2019-07-29

**Authors:** Soraia I. Falcão, Ricardo C. Calhelha, Soumaya Touzani, Badiaâ Lyoussi, Isabel C. F. R. Ferreira, Miguel Vilas-Boas

**Affiliations:** 1 Centro de Investigação de Montanha (CIMO), Instituto Politécnico de Bragança, Campus de Santa Apolónia, 5300-253 Bragança, Portugal; 2 Laboratory of Physiology, Pharmacology & Environmental Health, Faculty of Science, University Sidi Mohamed Ben Abdellah, Fez 30050, Morocco

**Keywords:** propolis, phenolic compounds, cytotoxicity, tumor cells, anti-inflammatory

## Abstract

Propolis is a resin manufactured by bees through the mixture of plant exudates and waxes with secreted substances from their metabolism, resulting in a complex mixture of natural substances of which quality depends on the phytogeographic and climatic conditions around the hive. The present study investigated the contribution of phenolic compounds to the cytotoxic and anti-inflammatory activities of propolis. The phenolic composition was evaluated by liquid chromatography with diode-array detection coupled to electrospray ionization tandem mass spectrometry (LC/DAD/ESI-MS^n^) analysis after phenolic extraction. The cytotoxicity of the extracts was checked using human tumor cell lines (MCF7- breast adenocarcinoma, NCI-H460- non-small cell lung carcinoma, HeLa- cervical carcinoma, HepG2- hepatocellular carcinoma, and MM127- malignant melanoma), as well as non-tumor cells (a porcine liver primary culture-PLP2). The anti-inflammatory activity was assessed using the murine macrophage (RAW 264.7) cell line. The results showed a composition rich in phenolic acids, such as caffeic and p-coumaric acid, as well as flavonoids, such as pinocembrin, pinobanksin, and pinobanksin-3-O-butyrate. Samples MP2 from Sefrou and MP3 from Moulay Yaâcoub presented a high concentration in phenolic compounds, while MP1 and MP4 from Boulemane and Immouzzer Mermoucha, respectively, showed similar composition with low bioactivity. The higher concentration of phenolic compound derivatives, which seems to be the most cytotoxic phenolic class, can explain the pronounced antitumor and anti-inflammatory activity observed for sample MP2.

## 1. Introduction

Plants and plant-derived products have proven to be rich sources of natural compounds, inducing many applications as new pharmaceutical agents. Nowadays, more than 60% of anticancer compounds come from natural products or substances derived from them, confirming interesting effects on cell lines and murine models and yielding higher activity compared to synthetic compounds [1,2]. Additionally, the combination of toxic natural products and monoclonal antibodies or polymeric carriers can lead to more effective targeted therapies, enabling synergistic or additive effects, which allow lower treatment concentrations and avoid multi-drug resistance, commonly developed when single drug molecules are used [2,3,4]. Natural products or natural product-derivatives may provide such combinations for use in chemotherapy and chemoprevention [3]. In recent years, modified natural products produced by bees, such as propolis, have been receiving great attention due to its wide spectrum of biological and pharmaceutical properties [2].

Propolis is a bee product that worker honey bees (*Apis mellifera* L.) prepare from resins that they collect from different plants and mix with beeswax. In some circumstances, bees may also cut fragments of vegetative tissues to release the resin used in propolis production [5]. Bees make use of propolis as a building material but also to defend the hive against pathogens [6]. It plays an important role in the colony-level immunity defense since the sources of the resin are complex plant secretions with high antimicrobial properties [7]. The chemical variability of propolis is strongly reliant on the plant sources available around the hive, which depends on the geographic and climatic conditions of the site, although bees show some preference for specific resin sources. This preference seems to be linked to the sticky and resinous properties of the material, but there is also some evidence that bees select plant resins rich in biologically active phytochemicals that fulfill their needs [5,7].

Propolis contains approximately 50% resin and vegetable balsam (phenolic compounds), 30% of wax, 10% essential and aromatic oils, 5% pollen, and 5% other substances (amino acids, vitamins, mineral salts), including organic debris [8,9]. It is known to possess valuable biological properties such as antimicrobial, anti-inflammatory, antitumor and antioxidant activities, which are due to its rich composition in phenolic compounds [10,11]. Within the most abundant compounds, we may find flavonoids such as pinocembrin, pinobanksin, galangin and chrysin, common in propolis from temperate regions where poplar are the main source of the resin, while prenylated phenylpropanoids, like artepillin C, are found in Brazilian propolis with origin in *Baccharis* spp [5,12]. Numerous scientific works have focused their research on propolis phenolic compounds as a potential tool for the development of new drugs to use against cancer [10]. Several mechanisms of action against tumor cells are described for propolis phenolic extracts, including induction of apoptosis, cell-cycle arrest, inhibition of matrix metalloproteinases, anti-angiogenesis effect, and prevention of metastasis and invasion [2,10,13,14].

Previous studies on Moroccan propolis extracts were shown to have in vitro and in vivo antitumor potential against three mammalian tumor cell lines: BSR (hamster renal adenocarcinoma), Hep-2 (human laryngeal carcinoma) and P815 (murin mastocytoma) [15]. Other biological activities such as antioxidant, antimicrobial [16], anti-inflammatory and acetylcholinesterase inhibitory activities [17] were also reported. Biological properties research of such a complex matrix as propolis should always be accompanied by chemical composition characterization of its botanical source, in order to explore its bioactivity and interaction with its constituents. Therefore, the aim of this study was to investigate the contribution of Moroccan propolis phenolic compounds to the bioactive properties of Moroccan propolis, in particular the anti-inflammatory activity and the cytotoxicity on human tumor cell lines (MCF-7—breast adenocarcinoma, NCI-H460—non-small cell lung carcinoma, HeLa—cervical carcinoma, HepG2—hepatocellular carcinoma, MM127—human malignant melanoma) and non-tumor primary cells (PLP2).

## 2. Materials and Methods

### 2.1. Standards and Reagents

Caffeic acid, *p*-coumaric acid, pinocembrin, chrysin, and caffeic acid phenylethyl ester (CAPE) were purchased from Sigma Chemical Co (St Louis, MO, USA). Kaempferol and apigenin were acquired from Extrasynthese (Genay, France). HPLC-grade methanol, ethanol, and acetonitrile were purchased from Fisher Scientific (Leics, UK). Water was treated in a Milli-Q water purification system (TGI pure system, Houston, TX, USA).

Fetal bovine serum (FBS), L-glutamine, Hank’s balanced salt solution (HBSS), trypsin- ethylenediaminetetraacetic acid (EDTA), penicillin/streptomycin solution (100 U/mL and 100 mg/mL, resp.), RPMI-1640, and DMEM media were purchased from Hyclone (Logan, UT, USA). Acetic acid, ellipticine, dexamethasone, sulforhodamine B (SRB), trypan blue, trichloroacetic acid (TCA), and tris were obtained from Sigma Chemical Co. (Saint Louis, MO, USA).

### 2.2. Propolis Samples

The study was performed on propolis samples collected, by scratching, from four different geographical locations, within the Fez region in Morocco: MP1 is from Boulemane (main vegetation: *Bupleurum*, *Ceratonia, Eucalyptus*, *Thymus* and *Rosmarinus*), MP2 from Sefrou (main vegetation: *Olea*, *Pinus*, *Quercus*, *Juniperus*, *Rosmarinus*, *Cistus*, *Lavandula* and *Pistacia*), MP3 from Moulay Yaâcoub (main vegetation: *Ceratonia*, *Citrus*, *Eucalyptus* and *Silybum*) and MP4 from Immouzzer Mermoucha (main vegetation: *Populus*, *Ceratonia*, *Eucalyptus*, *Rosmarinus* and *Quercus*). All the samples, with a similar brownish colour, were gathered in 2018 and kept at −20 °C until analysis.

### 2.3. Propolis Phenolic Compounds Extraction

The phenolic compounds were obtained through a hydro-ethanolic extraction procedure [11]. Briefly, 1 g of sample was mixed with 10 mL of 80% of ethanol/water and kept at 70 °C for 1 h under stirring. The resulting mixture was filtered and re-extracted in the same conditions. Finally, the resulting extracts were combined, concentrated, and freeze-dried.

### 2.4. Chemical Characterization of the Samples by LC/DAD/ESI-MS^n^

The LC/DAD/ESI-MS^n^ analyses were performed on a Dionex Ultimate 3000 UPLC instrument (Thermo Scientific, San Jose, CA, USA) equipped with a diode-array detector and coupled to a mass detector. The column used for high-performance liquid chromatography (HPLC) was a Macherey-Nagel Nucleosil C18 (250 mm × 4 mm id; 5 mm particle diameter, end-capped) and its temperature was maintained at 30 °C. The LC conditions used followed our previous work [12]. A flow rate of 1 mL/min was applied; with an injection volume of 10 µL. Spectral data for all peaks were accumulated in the range of 190–600 nm.

The mass spectrometer was operated in the negative ion mode using Linear Ion Trap LTQ XL mass spectrometer (Thermo Scientific, CA, USA) equipped with an electrospray ionization (ESI) source. ESI source parameters were as follows: Source voltage, 5 kV; capillary voltage, −20 V; tube lens voltage, −65 V; capillary temperature, 325 °C; and sheath and auxiliary gas flow (N_2_) set as 50 and 10 (arbitrary units), respectively. Mass spectra were acquired on full range acquisition covering 100–1000 m/z. For the fragmentation study, a data dependent scan was performed by deploying collision-induced dissociation (CID). The normalized collision energy of CID cell was set at 35 (arbitrary units). Data acquisition was carried out with Xcalibur^®^ data system (Thermo Scientific, San Jose, CA, USA).

The elucidation of the phenolic compounds was achieved by comparison of their chromatographic behavior, UV spectra, and MS information, to those of reference compounds. When standards were not available, the structural information was confirmed with UV data combined with MS fragmentation patterns previously reported in the literature.

Quantification was achieved using calibration curves for caffeic acid (0.002–0.35 mg/mL; y = 3 × 10^7^x − 78,726; R^2^ = 0.999), p-coumaric acid (0.02–0.15 mg/mL; y = 5 × 10^7^x − 94,095; R^2^ = 0.999), chrysin (0.0025–0.16 mg/mL; y = 2 × 10^7^x − 7021; *R^2^* = 0.999), kaempferol (0.005–0.075 mg/mL; y = 6 × 10^6^x − 2761; *R^2^* = 0.999), and pinocembrin (0.005–1 mg/mL; y = 2 × 10^7^x − 247,019; R^2^ = 0.999). When the standard was not available, the compound quantification was expressed in equivalent terms of the structurally closest compound. The assays were performed in duplicate and the results expressed as mg/g of extract.

### 2.5. Cytotoxic Activity

The cytotoxic effects were evaluated in five human tumor cell lines: MCF-7 (breast adenocarcinoma), NCI-H460 (non-small cell lung cancer), HeLa (cervical carcinoma) and HepG2 (hepatocellular carcinoma), obtained from DSMZ (Leibniz-Institut DSMZ–Deutsche Sammlung von Mikroorganismen und Zellkulturen GmbH), and MM127 (human malignant melanoma) from the ECACC General Cell Collection. All the cells were routinely maintained as adherent cell cultures in RPMI-1640 medium, containing 10% FBS, 2 mM glutamine, 100 U/mL penicillin, and 100 µg/mL streptomycin, and kept at 37 °C in a humidified air incubator containing 5% CO_2_. The cytotoxic potential of the propolis samples was evaluated by the sulforhodamine B (SRB) assay, according to the procedure described previously [18]. For hepatotoxicity evaluation, a cell culture was prepared from a freshly harvested porcine liver obtained from a local slaughterhouse, according to an established procedure, and designated as PLP2 (porcine liver primary culture). The cell cultivation was maintained, with direct monitoring, every two to three days using a phase contrast microscope. Before confluence was reached, cells were subcultured and plated in 96-well plates at a density of 1.0 × 10^4^ cells/well, and cultivated in DMEM medium with 10% FBS, 100 U/mL penicillin, and 100 µg/mL streptomycin. The propolis samples were dissolved in 50% DMSO:water to obtain a stock solution of 10 mg/mL. Cells were treated with different concentrations of the samples (400–6.25 µg/mL) and SRB assay was performed as previously described. The results were expressed as GI_50_ values in µg/mL (sample concentration that inhibited 50% of the net cell growth). Ellipticine was used as a positive control.

### 2.6. Anti-Inflammatory Activity

The anti-inflammatory activity was evaluated using the murine macrophage (RAW 264.7) cell line, obtained from ECACC General Cell Collection. Cells were routinely maintained as adherent cell cultures in DMEM medium containing 10% FBS, 2 mM glutamine, 100 U/mL penicillin, and 100 µg/mL streptomycin, and kept at 37 °C in a humidified air incubator containing 5% CO_2_ [19]. The extracts were dissolved in DMSO/water (1:1) obtaining a stock solution with a final concentration of 10 mg/mL. Nitric oxide (NO) production was studied with a Griess Reagent System kit. Results were expressed as IC_50_ values (μg/mL), equal to the sample concentration providing a 50% inhibition of NO production. The cells treatment and the nitric oxide determination was carried out as previously described [19] and compared to the standard calibration curve (y = 0.073 + 0.1509; *R^2^* = 0.999). Dexamethasone was used as a positive control.

### 2.7. Statistical Analysis

All experiments were performed in duplicate. Results are expressed as mean values and standard deviation (SD). The differences between samples were checked using analysis of variance (ANOVA) followed by Tukey post hoc significant difference test with α = 0.05. Additionally, a multivariate statistical analysis (Spearman rank correlation analysis) was performed to establish links between propolis chemical composition and the biological activities, and to assess the relationship between variables. The statistical analysis was performed using the program SPSS v. 20.0.

## 3. Results and Discussion

### 3.1. Moroccan Propolis Chemical Characterization

The chemical complex nature of propolis is linked to the resinous materials gathered by honey bees from different floral sources available around the hive, which have a direct impact on the quality and bioactivity of the propolis. Liquid chromatography coupled with mass spectrometry is a powerful tool that can be used to overcome the difficult task of propolis chemical profiling [6].

The phenolic composition of the four propolis samples from Morocco was evaluated by LC/DAD/ESI-MS^n^, with the identification of twenty-six phenolic compounds. Negative ion mode was used in this study because of its higher analytical sensitivity for different polyphenol classes [12]. Caffeic acid, *p*-coumaric acid, pinobanksin, pinocembrin, chrysin, and pinobanksin-3-*O*-acetate (Table 1) were present in all of the samples, and most likely arose from the common source of the resin, the poplar tree. However, other species like *Ulmus*, *Pinus*, *Quercus*, *Salix* e *Acacia*, can be also adequate sources for temperate propolis, particularly when poplars are unavailable [5]. Some compounds were specific to only one sample, such as sterubin, 3-prenyl-*p*-coumaric acid, dihydrokampferide, capillartimisin A, and isosakuranetin found in sample MP4 from Immouzzer Mermoucha; pinobanksin-5-methyl-ether (3, *m/z* 285) present in sample MP1 from Boulemane; apigenin, caffeic acid pentyl ester, pinobanksin-3-*O*-propionate, pinobanksin-3-*O*-butyrate, and pinobanksin-3-*O*-pentanoate observed in MP2 from Sefrou; and benzoyl hydroxyphenyl acetic acid only present in MP3 from Moulay Yaâcoub. Phenolic acid derivatives such as caffeic acid isoprenyl ester, caffeic acid benzyl ester, and caffeic acid phenylethyl ester (CAPE) were observed in samples MP1, MP2, and MP3, while flavonols such as kaempferol-methyl-ether, galangin and 6-methoxychrysin were characteristic of MP2 and MP3 samples (Table 1). Besides the compounds mentioned above, the composition of samples MP1 and MP4 included a chemical structure with *m/z* 377, which was not possible to identify, and therefore described as unknown (Table 1). Overall, the phenolic composition of the Moroccan propolis resembles other studies from the group [20], with a resin rich in flavonoid aglycones and phenolic acid esters with similarities to the poplar propolis.

**Figure 1:**
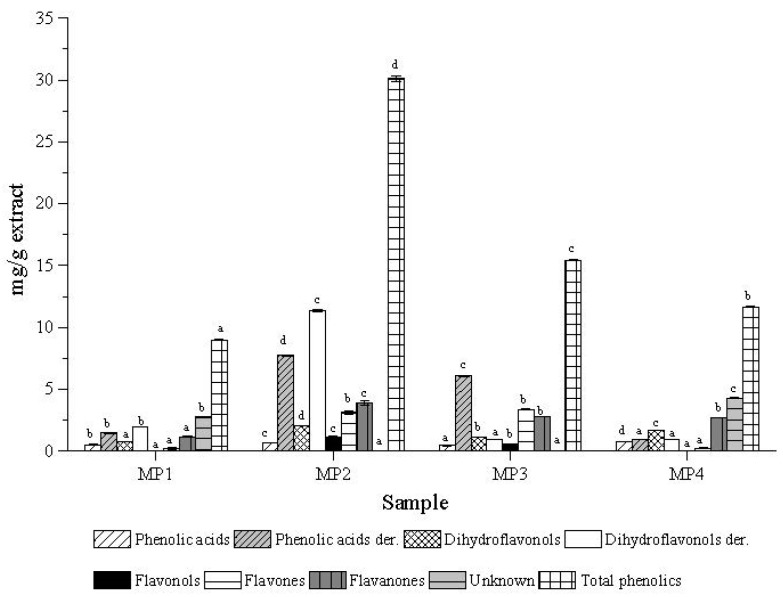
Principal phenolic classes presented in the composition of propolis from different Morocco origins.

Figure 1 displays the quantification analysis for the different samples. There is an evident difference between samples MP2 (30 mg/g of total phenolics) and MP3 (15mg/g of total phenolics), with MP2 containing twice the amount of phenolics. Samples MP1 and MP4 presented the poorest phenolic profile, with only twelve compounds identified and a total phenolic content of 9 and 12 mg/g extract, respectively.

In respect to the individual classes, dihydroflavonol derivatives, amongst them pinobanksin-3-*O*-acetate, were the main compounds present in these Moroccan propolis samples, especially in sample MP2. This was followed by the phenolic acids derivatives, where the phenolic acids esters emerge as the major compounds (Figure 1). Flavonols, such as kaempferol-methyl-ether and galangin were found as minor compounds, present only in samples MP2 and MP3 (Table 1).

### 3.2. Cytotoxicity and Anti-Inflammatory Activity

**Figure 2:**
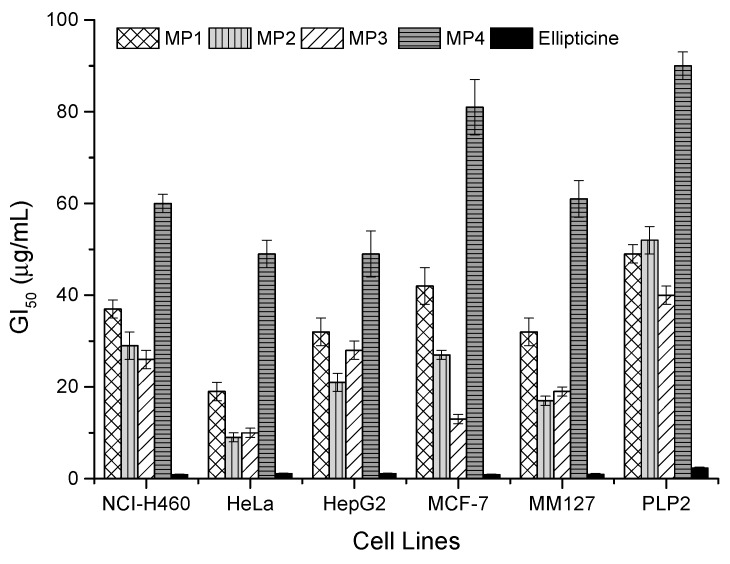
Cytotoxic activity of propolis samples on cell lines expressed in GI_50_ values (µg/mL) (mean ± SD, *n* = 2). GI_50_ values correspond to the extract concentration achieving 50% of growth inhibition in human tumor cell lines or in liver primary culture PLP2.

The chemical compounds presented in the resin, which bees collect from plants to prepare propolis, are, in general, powerful bioactive metabolites used by plants for protection against pathogens, and so, are used by bees to immunize the hive environment [13]. Therefore, they may also function well as a relevant source of bioactive substances for pharmaceutical purposes. With this in mind, the cytotoxicity of the Moroccan propolis phenolic extracts was tested in five human tumor cell lines, including MCF-7 (breast adenocarcinoma), NCI-H460 (non-small cell lung cancer), HeLa (cervical carcinoma), HepG2 (hepatocellular carcinoma), and MM127 (human malignant melanoma) as well as in a porcine liver primary cell culture (PLP2), established by us. Also, the anti-inflammatory activity was evaluated using the murine macrophage (RAW 264.7) cell line. The growth inhibition of the tumor cells was observed in all the experiments, but particularly on sample MP2, which had the lowest cytotoxicity GI_50_ values in three of the five cell lines (Figure 2), followed by MP3 with the best performance for breast adenocarcinoma and lung cancer cells.

Overall, growth inhibition GI_50_ results were lower compared to the ones reported by us in Portuguese propolis [25]. The activity against HeLa cell line is particularly interesting for samples MP2 and MP3, with GI_50 values_ of 9 μg/mL and 10 μg/mL, respectively (Figure 2), which is not as far from the activity of the synthetic standard elliptimicine as we would expect for a mixture of natural substances. On the opposite side is the sample MP4 from Immouzzer, with the weakest performance against all the tumor cell lines tested. Although it shows a richer composition in total phenolic compounds than MP1, the poor performance can be explained by the low concentration of specific bioactive compounds, such as those derived from the phenolic acids. Regardless of the high cytotoxicity exhibited by the Moroccan propolis against the studied tumor cell lines, all the extracts displayed some toxicity for non-tumor (normal) liver primary culture (PLP2). However, the values obtained for the tumor cells were always below the ones for non-tumor cells, highlighted in the results obtained for HeLa, MCF-7 and MM127, especially in the case of samples MP2 and MP3 (Figure 2).

The reported bioactivity is most probably correlated with the phenolic composition of propolis. Indeed, MP2 and MP3 as the samples with higher phenolic content were the ones with higher cytotoxicity. Several mechanisms of action are described for the interaction of phenolic compounds with tumor cells, including induction of apoptosis, cell-cycle arrest, inhibition of matrix metalloproteinases, anti-angiogenesis effect, and prevention of metastasis and invasion [2,10,13,14].

From Table 2 it is possible to retrieve additional information with respect to the correlation between the bioactivity against the different cell lines, and the phenolic classes. Without any doubt, the phenolic acid derivatives are the most active class, exhibiting a negative correlation (higher cytotoxicity) for all the cell lines, and particularly against HeLa and MM127.

This class of compounds, which includes phenolics such as caffeic acid phenylethyl ester (CAPE), observed in higher amounts in samples MP2 and MP3, is reported to play a key role in the bioactivity of propolis extracts, including against rat glioma C6, human squamocosal carcinoma, breast murine B16F10, human breast adenocarcinoma, and different leukemia cells [26]. The cytotoxicity of flavones seems to be more selective, with greater correlation to lung and hepatocellular carcinoma (Table 2). Indeed, compounds such as chrysin, found in higher amounts in propolis, have been shown to act against Murine B16F10, human colon carcinoma HCT-15 and human hepatoma Hep-3B cells [14].

One of the classes most represented in the Moroccan propolis samples under study, the dihydroflavonols derivatives, seems to correlate only with cervical carcinoma HeLa and malignant melanoma, MM127. These findings corroborate previous studies on Moroccan propolis extracts, which also observed an anti-proliferative effect against other skin tumor cells such as P815 (murine mastocytoma), in addition to effects against BSR (hamster renal adenocarcinoma) and Hep-2 (Human laryngeal carcinoma) [15]. Recently, dihydroflavonols such as pinobanksin and its derivatives, for example pinobanksin-3-*O*-acetate, were described to exert an anti-proliferative effect, induced through apoptosis in a B-cell lymphoma cancer cell line [27]. Furthermore, it was reported that the anti-metastatic effectiveness of the entire propolis extract was higher than that presented by its individual constituents, resulting in a synergistic effect between constituents [13]. Overall, these compounds are described to target various genetic and biochemical pathways of cancer progression like induction of apoptosis, cell-cycle arrest, inhibition of matrix metalloproteinases, anti-angiogenesis effect, and prevention of metastasis and invasion [10,14,26].

**Figure 3:**
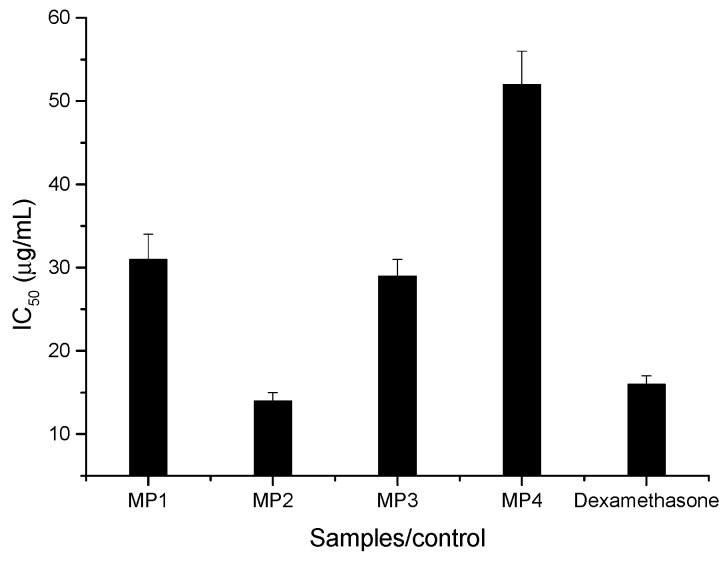
Anti-inflammatory activity of propolis samples expressed in IC_50_ values (µg/mL) of NO production inhibition (mean ± SD, *n* = 2). IC_50_ values correspond to the extract concentration achieving 50% of the inhibition of the NO production.

The anti-inflammatory effect, whose results are summarized in Figure 3, was assessed using murine macrophage-like RAW 264.7 cells and quantified through the nitric oxide (NO) production.

All propolis extracts under study showed anti-inflammatory capacity, with IC_50_ values between 14 and 52 µg/mL. The highest activity was observed for sample MP2, which contains a higher quantity of bioactive compounds such as phenolic acid derivatives and flavones, followed by the sample MP3, with an IC_50_ value of 29 µg/mL. It is worth mentioning the great performance of sample MP2, which displayed an even higher anti-inflammatory activity than the positive control dexamethasone. Compounds such as phenolic acid derivatives, flavonols, and dihydroflavonols derivatives, seem to be the major responsible substances, as considering their significant negative correlation with anti-inflammatory activity (Table 2). This also may explain the fact that, despite the low concentration of phenolic compounds in sample MP1, its anti-inflammatory activity was similar to that of sample MP3, which can be attributed to the higher concentration of dyhidroflavonols derivatives in the chemical composition of sample MP1 as compared to sample MP3 (Figure 1).

## 4. Conclusions

Four samples of Moroccan propolis from the Fez region were evaluated to determine their phenolic composition, cytotoxicity against tumor cell lines, and anti-inflammatory activity. The data presented here indicates that Moroccan propolis is composed of a complex phenolic composition, with 26 phenolic compounds identified. According to the cytotoxicity analysis, Moroccan propolis presents high toxicity against tumor cell lines, in particular HeLa (cervical carcinoma) and MM127 (human malignant melanoma). Compounds such as phenolic acid derivatives and flavonols seem to be the phenolic classes that contribute most to the cytotoxic effect. In some cases, the closeness of the in vitro doses for tumor and normal cell lines should be taken into consideration and confirmed by in vivo tests. Additionally, the propolis extracts displayed high anti-inflammatory activity, which was evaluated using murine macrophage-like RAW 264.7 cells, and the activity explained through the presence of phenolic acid and dihydroflavonols derivatives. Further studies concerning the isolation of major phenolic compounds and its biological properties against different tumor and non-tumor cell lines should be investigated. Also, phenolic compounds are described to have different mechanisms of action against tumor cells—they can target human cellular receptors, enzymes, transcription factors, and DNA. Future work with these samples will complement this study and develop further understandings of the mechanism of action of phenolic compounds in anticancer treatment. Moreover, propolis action should be compared to antitumor drugs, or even be tested in association with them, to investigate a possible synergistic action. These results support the hypothesis that specific phenolic compounds contribute greatly to the bioactive properties of propolis.

## Figures and Tables

**Table 1:** Phenolic compounds in Moroccan propolis identified and quantified by LC/DAD/ESI-MS^n^ (each value is the mean ± standard deviation, *n* = 2).

Peak	Proposed Compound	RT (min)	λ_max_ (nm)	[M − H]^−^ *m/z*	MS^n^ (% Base Peak)	MP1 mg/g Extract	MP2 mg/g Extract	MP3 mg/g Extract	MP4 mg/g Extract
1	Caffeic acid ^a,b^	10.9	322	179	MS^2^ [179]: 135	0.31 ± 0.00	0.50 ± 0.00	0.31 ± 0.00	0.30 ± 0.00
2	*p*-Coumaric acid ^a,b^	15.8	310	163	MS^2^ [163]: 119	0.22 ± 0.00	0.21 ± 0.00	0.16 ± 0.00	0.50 ± 0.00
3	Pinobanksin-5-methyl-ether ^b,c^	36.9	286	285	MS^2^ [285]: 267 (100), 253 (13), 239 (27)	0.77 ± 0.01			
4	Sterubin ^b,d^	47.3	284	301	MS^2^ [301]: 286 (100), 165 (8); MS^3^ [286]: 258 (37), 195 (17), 165 (100)				0.83 ± 0.01
5	Pinobanksin ^b,c^	47.3	292	271	MS^2^ [271]: 253 (100), 225 (26), 151 (10)	0.83 ± 0.00	2.04 ± 0.00	1.14 ± 0.03	0.83 ± 0.01
6	Dihydrokaempferide ^b,e^	50.4	291	301	MS^2^ [301]: 283 (100), 151 (22); MS^3^ [283]: 268 (100), 255 (40), 227 (41)				0.86 ± 0.01
7	Apigenin ^a,b^	54.9	268, 337	269	MS^2^ [269]: 225 (100), 151 (29)		0.27 ± 0.00		
8	Kaempferol-methyl ether ^b,c^	57.7	265, 352	299	MS^2^ [299]: 284		0.15 ± 0.01	0.26 ± 0.00	
9	3-prenyl-*p*-coumaric acid ^b,e^	63.6	315	231	MS^2^ [231]: 187; MS^3^ [187]: 132				0.71 ± 0.00
10	Caffeic acid isoprenyl ester ^a,b^	65.2	325	247	MS^2^ [247]: 179 (100), 135 (13)	0.34 ± 0.00	1.73 ± 0.01	1.40 ± 0.00	
11	Caffeic acid isoprenyl ester ^b,c^	66.3	325	247	MS^2^ [247]: 179 (100), 135 (13)	0.35 ± 0.01	3.00 ± 0.01	2.59 ± 0.00	
12	Capillartimisin A ^b,e^	66.4	309	315	MS^2^ [315]: 285 (60), 271 (100), 241 (67); MS^3^ [271]: 253 (41), 241 (100)				0.21 ± 0.00
13	Caffeic acid benzyl ester ^b,c^	66.7	325	269	MS^2^ [269]: 178 (100), 161 (12), 134 (32)	0.43 ± 0.01	1.18 ± 0.03	0.90 ± 0.02	
14	Pinocembrin ^a,b^	67.6	289	255	MS^2^ [255]: 213 (100), 211 (32), 151 (48)	1.17 ± 0.04	3.91 ± 0.15	2.80 ± 0.03	1.81 ± 0.00
15	Isosakuranetin ^b,f,g^	68.1	292	285	MS^2^ [285]: 270 (100), 243 (25), 164 (17), 151 (4) MS^3^ [270]: 242 (41), 165 (100), 164 (70)				0.92 ± 0.00
16	Benzoyl hydroxyphenyl acetic acid ^b,f^	68.4	280	257	MS^2^ [257]: 213; MS^3^ [213]: 169 (100), 122 (49)			0.61 ± 0.02	
17	Chrysin ^a,b^	69.6	268, 313	253	MS^2^ [253]: 225 (17), 209 (100), 151 (5)	0.25 ± 0.08	1.99 ± 0.14	2.88 ± 0.01	0.27 ± 0.03
18	Pinobanksin-3-*O*-acetate ^b,c^	69.6	292	313	MS^2^ [313]: 271 (20), 253 (100)	1.22 ± 0.02	6.82 ± 0.06	1.01 ± 0.00	0.96 ± 0.02
19	Caffeic acid phenylethyl ester ^a,b^	69.9	325	283	MS^2^ [283]: 179 (100), 135 (22)	0.37 ± 0.02	1.24 ± 0.03	0.57 ± 0.00	
20	Galangin ^a,b^	70.3	265, 300sh, 358	269	MS^2^ [269]: 269 (100), 241 (61), 227 (20), 197 (22), 151 (20)		1.04 ± 0.02	0.35 ± 0.00	
21	Caffeic acid pentyl ester ^b^	71.1	325	249	MS^2^ [249]: 179 (100), 161 (47), 135 (32)		0.62 ± 0.00		
22	6-Methoxychrysin ^b,c^	72.3	265, 300sh, 350sh	283	MS^2^ [283]: 269		0.88 ± 0.05	0.48 ± 0.04	
23	Pinobanksin-3-*O*-propionate ^b,c^	75.2	289	327	MS^2^ [327]: 271 (9), 253 (100)		1.08 ± 0.01		
24	Unknown	79.5	239	377	MS^2^ [377]: 359 (100), 331 (84), 313 (8)	2.76 ± 0.04			4.31 ± 0.03
25	Pinobanksin-3-*O*-butyrate or isobutyrate ^b,c^	79.9	292	341	MS^2^ [341]: 271 (2), 253 (100)		2.66 ± 0.01		
26	Pinobanksin-3-*O*-pentanoate or 2-methylbutyrate ^b,c^	84.1	292	355	MS^2^ [355]: 271 (3), 253 (100)		0.80 ± 0.00		

^a^ Confirmed with standard; ^b^ Confirmed with MS^n^ fragmentation; ^c^ Confirmed with references: [12]; ^d^ [21]; ^e^ [22]; ^f^ [23]; ^g^ [24].

**Table 2:** Spearman’s correlations between the propolis phenolic composition (mg/g of extract) and cytotoxicity and anti-inflammatory activity GI_50_/IC_50_ values (μg/mL).

	NCI-H460	HeLa	HepG2	MCF-7	MM127	PLP2	RAW264.7
Phenolic acids	0.786 ^a^	0.381	0.786 ^a^	0.452	0.357	0.881 ^a^	0.357
Phenolic acid derivatives	−0.810 ^a^	−0.976 ^b^	−0.762 ^a^	−0.833 ^a^	−0.952 ^b^	−0.500	−0.905 ^b^
Dihydroflavonols	0.024	−0.381	0.024	−0.310	−0.357	0.429	−0.381
Dihydroflavonols derivatives	−0.405	−0.810 ^a^	−0.357	−0.619	−0.738 ^a^	−0.310	−0.857 ^b^
Flavonols	−0.710 ^a^	−0.913 ^b^	−0.710 ^a^	−0.862 ^b^	−0.888 ^b^	−0.317	−0.875 ^b^
Flavones	−0.881 ^b^	−0.619	−0.881 ^b^	−0.690	−0.690	−0.548	−0.500
Flavanones	−0.619	−0.786 ^a^	−0.571	−0.643	−0.762 ^a^	−0.167	−0.667
Total phenolics	−0.595	−0.762 ^a^	−0.548	−0.667	−0.786 ^a^	−0.143	−0.643

Spearman’s correlation significance levels: ^a^ Correlation is significant at *p* < 0.05; ^b^ Correlation is significant at *p* < 0.01.
